# Fractal Characteristic-Induced Optimization of the Fixed Abrasive Lapping Plate in Fabricating Bipolar Plate of Proton-Exchange Membrane Fuel Cells

**DOI:** 10.3390/ma15175922

**Published:** 2022-08-26

**Authors:** Guoqing Pan, Zhengwei Wang, Donghui Wen

**Affiliations:** 1 Special Equipment Institute, Hangzhou Vocational & Technical College, Hangzhou 310018, China; 2 College of Mechanical Engineering, Zhejiang University of Technology, Hangzhou 310023, China

**Keywords:** fixed abrasive lapping, bipolar plate, fractal characteristics, uniform evaluation, box-counting dimension

## Abstract

*Purpose*: A bipolar plate with fractal-characterized microstructures can realize intelligent energy transmission and obtain a high efficiency of proton-exchange membrane fuel cells. In this paper, fixed abrasive lapping technology is proposed to fabricate a surface microstructure on a bipolar plate with fractal characteristics. *Methodology*: The kinematics of the fixed abrasive lapping process was developed and employed to numerically investigate the particle trajectories moving on the target surface by considering the different arraying forms of diamonds on the lapping plate. *Findings*: It was found from an analysis of both the uniformity and the fractal characteristics that the arraying form of diamonds on the lapping plate, with the distribution of latitude and longitude with an angle of 30° and a gap of concentric circles of 40 mm with a minimum radius of 70 mm and maximum radius of 190 mm, can be used to obtain the best uniformity and fractal characteristics in the fixed abrasive lapping of a bipolar plate. *Conclusions*: The distribution of the latitude and longitude of 40° and 30° considered in this study is expected to realize the best machining performance in the bipolar plate and present good cell performance.

## 1. Introduction

In nature there are special geometric structures in the plant trunk, leaf vein, and blood circulation system [1,2]. These geometric structures have a uniform distribution of microtubules, which can automatically adjust the structure of the flow field according to the growth, divergence, or degradation of working conditions, subsequently realizing the intelligent and biological flow through the assistance of these geometric structures [3]. It is also interesting to note from these geometric structures that they generally have fractal characteristics, which can be considered as the structure of the flow field in a bipolar plate to realize intelligent energy transmission and obtain a high efficiency of proton-exchange membrane fuel cells (PEMFCs). Thus, it is necessary to investigate the highly efficient fabrication of fractal-characterized bipolar plates for PEMFCs [4].

**Figure 1:**
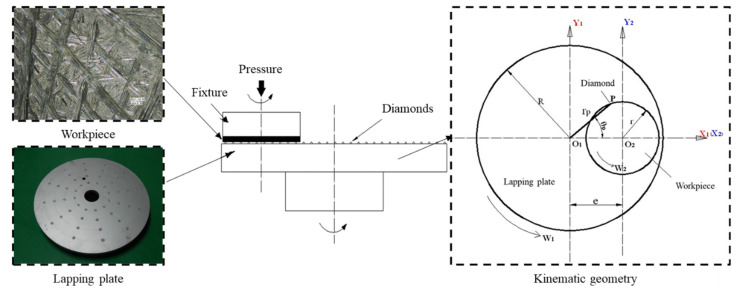
Schematic of the fixed abrasive lapping process.

How to fabricate a bipolar plate with fractal-characterized structures has attracted the attention of many researchers. Various techniques for forming metallic bipolar plates, such as electrochemical micromachining process [5], magnetic pulse forming [6], additive manufacturing [7], and high-vacuum die casting [8], have been proposed to fabricate bipolar plates, but they are either time-consuming or expensive. Abrasive machining technology, as a nontraditional machining technology, has been extensively used in the fabrication of microstructures with almost any material [9,10,11,12], such as the abrasive slurry jet micromachining of micro-holes and channels [13] and the liquid metal lapping–polishing of free-form surface structures [14,15], in which the fixed abrasive lapping technology seems to be an attractive avenue to be explored for meeting these pressing needs [16,17,18]. In the fixed abrasive lapping process, the diamonds are fixed in the lapping plate according to different arraying forms, and the relative motion between the lapping plate and workpiece allows realizing the fabrication of microgrooves on the target surface with a certain depth and width, as shown in Figure 1. These microgrooves can form the surface microstructure with fractal characteristics on the bipolar plate. Zhang et al. employed the fixed lapping tool to machine hard/brittle materials for surface/subsurface damage evaluation [19]. Sanchez et al. investigated the lapping kinematics on abrasive discs for finishing of flat workpieces using a combination of important grinding and lapping characteristics, and they revealed that the kinematics of the lapping plate and workpiece had an essential effect on the machining performance [20]. The mechanism of material removal by the fixed abrasive lapping of fused quartz glass was also studied by Lin et al. to increase the material removal rate, as well as reduce the subsurface damage [21]. However, these studies on fixed abrasive lapping technology were mainly on the surface integrity of the machined target, whereas limited research effort has been made on the optimized design of a fixed abrasive lapping plate that could be used to generate different surface structures on the target surface.

Thus, in this paper, different arraying forms of diamonds on the lapping plate were considered to explore the optimized design of a fixed abrasive lapping plate. The kinematics of the fixed abrasive lapping process was first theoretically developed to model the particle trajectory of any diamond on the target surface, and then a numerical simulation of the particle trajectories of many diamonds on target surface with respect to different arraying forms was carried out to qualitatively analyze their machining performance. Lastly, a quantitative analysis was conducted to explore the optimized design of the lapping plate by considering the uniformity and fractal characteristics of particle trajectories in the fixed abrasive machined target surfaces.

## 2. Kinematic Modeling of the Particle Trajectories on Target Surface in the Fixed Abrasive Lapping Process

The traditional abrasive lapping process faces challenges in machining the deterministic surface microstructure, such that, in order to ensure the machining of a deterministic surface microstructure on a bipolar plate, it is necessary to reasonably design the arrangement of diamonds on the fixed abrasive lapping plate. A reasonable arrangement can ensure the uniform particle trajectories on the target surface and confer these trajectories with fractal characteristics [18]. As such, after these particle trajectories move on the target, a deterministic surface microstructure with uniform and fractal characteristics can be generated, thereby realizing the machining of a bipolar plate with desire surface microstructure.

Since the formation of the surface microstructure on the target surface is caused by the relative motion between the diamonds on the lapping plate and the workpiece, it is first necessary to develop a kinematic model to explore the particle trajectories on the target surface in the fixed abrasive lapping process. Figure 1 shows the kinematic geometry of the fixed abrasive lapping process, where any point P on the lapping plate in the coordinate system *σ*_1_ = [*O*_1_, *x*_1_, *y*_1_] can be considered as the diamond with position of *P* (*r_p_*cos*θ_p_*, *r_p_*sin*θ_p_*), the distance between the *O*_1_ and *O*_2_ is *e*, and the rotation speeds of the lapping plate and workpiece are *w*_1_ and *w*_2_, respectively. Thus, in the coordinate system *σ*_2_ = [*O*_2_, *x*_2_, *y*_2_], the kinematic motion equation can be expressed as

(1) $$\left\{ \begin{array}{l} x_{1}=r_{p}\cos\left(\theta_{p}+w_{p}t\right)-e\\ y_{1}=r_{p}\sin\left(\theta_{p}+w_{p}t\right) \end{array}\right..$$

However, the coordinate system *σ*_2_ = [*O*_2_, *x*_2_, *y*_2_] conducts the rotary motion along the workpiece with a rotation speed of *w*_2_, and Equation (1) only describes the relative motion of *P* with respect to the static workpiece. Thus, the relative motion of any point *P* on the lapping plate with respect to the rotating workpiece can be derived from the kinematic and coordinate rotation transformation, which can be taken from

(2) $$\left\{ \begin{array}{l} x_{1}^{\prime }=r_{p}\cos\left(\theta_{p}+w_{1}t-w_{2}t\right)-e\cos\left(w_{2}t\right)\\ y_{1}^{\prime }=r_{p}\sin\left(\theta_{p}+w_{1}t-w_{2}t\right)+e\sin\left(w_{2}t\right) \end{array}\right..$$

Therefore, the kinematic model to present the particle trajectories moving on the target surface in the fixed abrasive lapping process can be developed from Equation (2), and then the different arraying forms of diamonds on the lapping plate can be numerically analyzed to explore the optimized design, as detailed below.

## 3. Numerical Modeling of the Particle Trajectories on Target Surface with Respect to Different Arraying Forms

**Figure 2:**
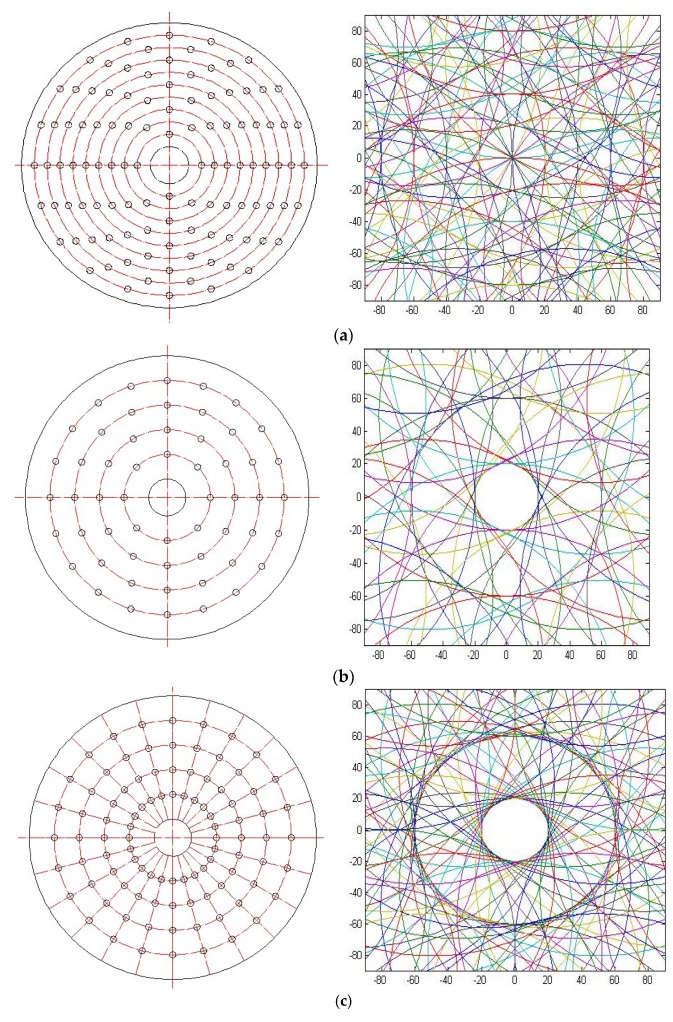

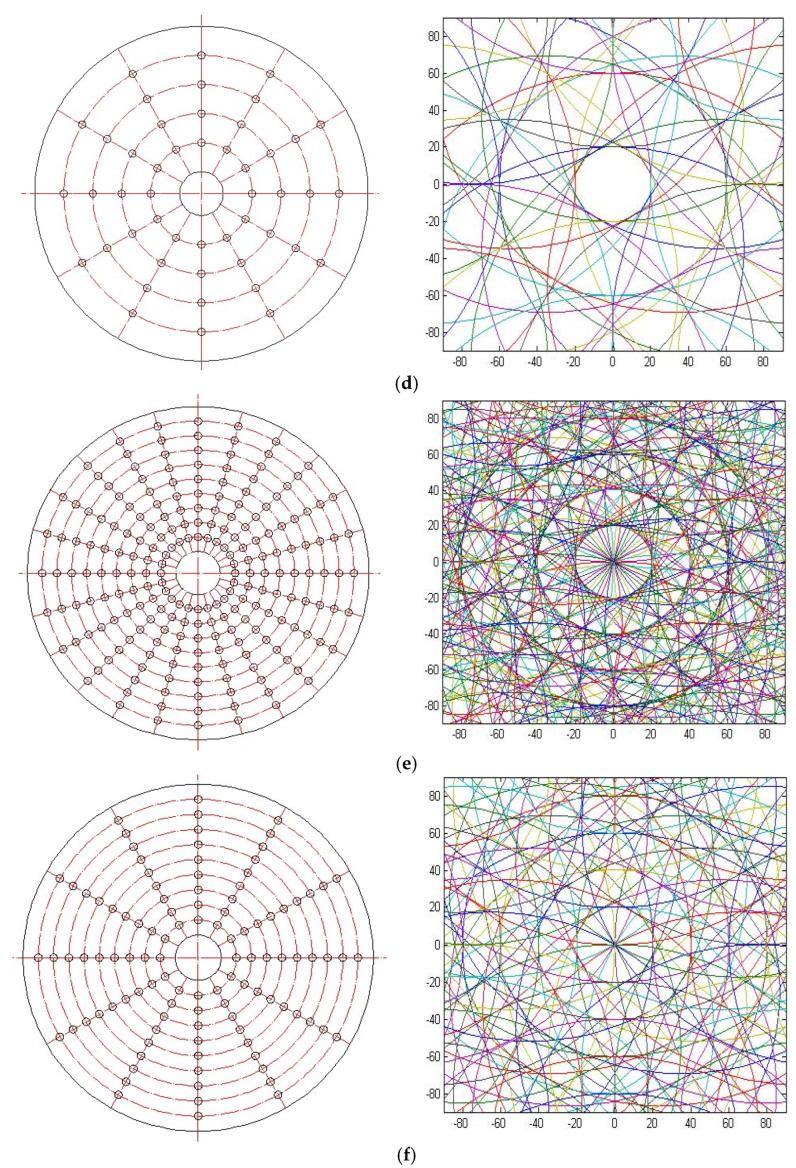
Schematics of different arraying forms of diamonds on the lapping plate and particle trajectories on target surface. (**a**) Circular arraying form: type-1. (**b**) Circular arraying form: type-2. (**c**) Latitude and longitude arraying form: 40°, 15°. (**d**) Latitude and longitude arraying form: 40°, 30°. (**e**) Latitude and longitude arraying form: 20°, 15°. (**f**) Latitude and longitude arraying form: 20°, 30°.

In order to explore the effects of different arraying forms of diamonds on the lapping plate on the distribution of particle trajectories moving on the target surface, in this study, six arraying forms were considered as given in Figure 2. Figure 2a,b show the circular arraying forms with a lapping plate radius of 230 mm, with different numbers of diamonds on concentric circles of different radii. Figure 2c,d present diamonds arranged on the lapping plate according to the distribution of the latitude and longitude, where the gap of the concentric circles is 40 mm with a minimum radius of 70 mm and a maximum radius of 190 mm. To be specific, in Figure 2c, the circle is divided into 24 grades with an angle of 15° between the diamonds, while, in Figure 2d, the circle is divided into 12 grades with an angle of 30° between the diamonds. Similarly, the gap of the concentric circles is 20 mm with a minimum radius of 50 mm and a maximum radius of 210 mm, while the circle is divided into 24 grades in Figure 2e and 12 grades in Figure 2f.

According to Equation (2) and the different arraying forms of diamonds on the lapping plate shown in Figure 2, the particle trajectories moving on the target surface can be numerically simulated in MATLAB, a powerful software to deal with different equations [22], which can be used to explore the ability of the fixed abrasive lapping of bipolar plates with desired surface microstructures. In the simulations, the processing parameters were set as follows: the diameter of the lapping plate was 230 mm, the radius of workpiece was 90 mm, the rotation speeds of lapping plate and workpiece were all 10 rad/min, and the lapping time was 6 min. Then, the simulation results were taken from the range of *x* [−90, 90] and *y* [−90, 90], as shown in Figure 2.

It can be seen from Figure 2 that the density of the particle trajectories moving on the target surface increased with an increase in the density of diamonds on the lapping plate, whereas Figure 2e shows the densest trajectory curves under latitude and longitude 20°, 15° distributed diamonds on the lapping plate. By comparing Figure 2a–d, it can be found that the latitude and longitude distribution of diamonds on the lapping plate would result in a petal-shaped distribution of particle trajectories on the target surface, whereas the circular arraying forms of diamonds on the lapping plate would lead to relatively messy distributions. Moreover, it is also interesting to note from Figure 2c–f that, in the latitude and longitude distribution of diamonds with a gap of concentric circles of 40 mm, the petal-shaped distribution of particle trajectories on the targe surface was more obvious than that with a gap of concentric circles of 20 mm. To sum up, all the arraying forms of diamonds on the lapping plate as discussed in this study could realize the particle trajectories moving on the target surface with fractal characteristics, such that it could result in the fixed abrasive lapping of bipolar plates with deterministic surface microstructures, but the optimized design is still necessary to be explored by quantitatively evaluating the uniformity of the particle trajectories on the target surface according to the material removal mechanisms caused by the abrasive [23,24,25].

## 4. Uniform Evaluation on the Distribution of Particle Trajectories on Target Surface

**Figure 3:**
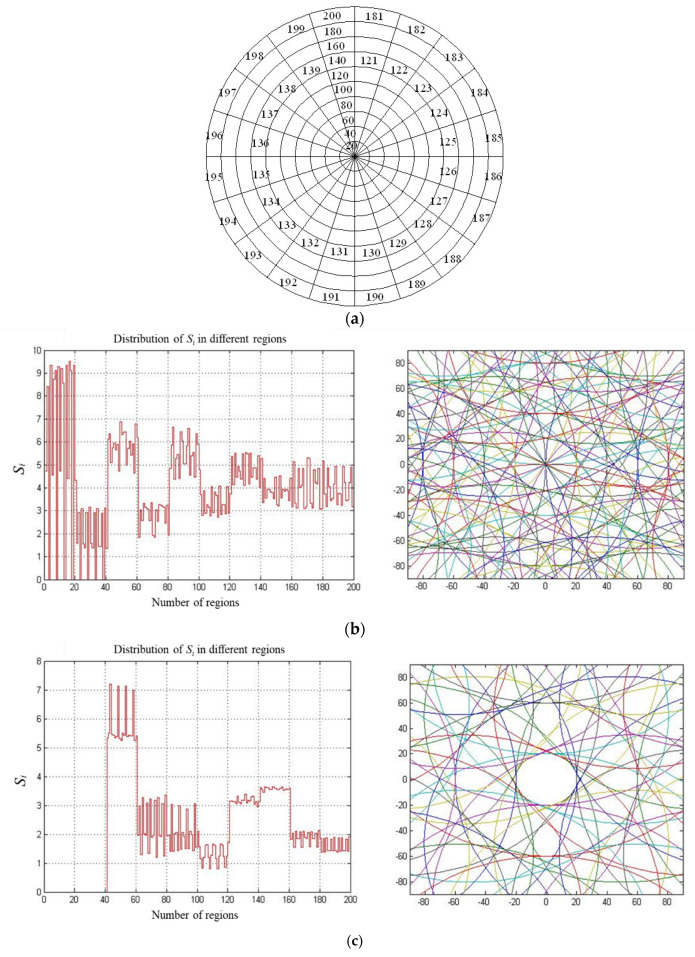

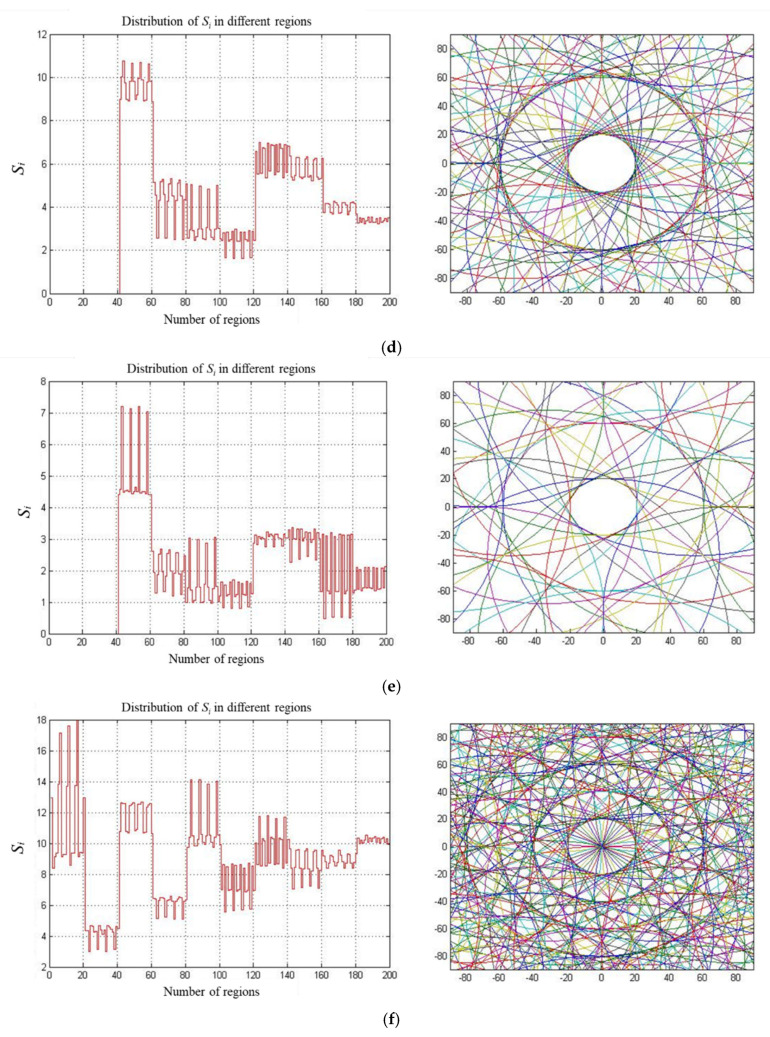

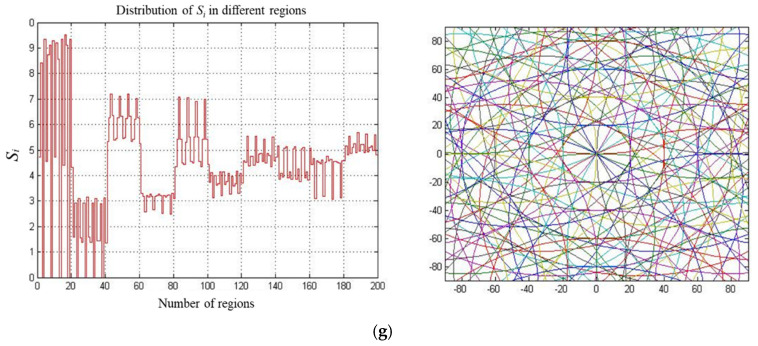
Distributions of *S_i_* in different regions and associated *S_T_* under different arraying forms. (**a**) Schematic of different regions divided on the target surface. (**b**) Circular arraying form: type-1, *S_T_* = 1.8623. (**c**) Circular arraying form: type-2, *S_T_* = 1.6727. (**d**) Latitude and longitude arraying form: 40°, 15°, *S_T_* = 2.8171. (**e**) Latitude and longitude arraying form: 40°, 30°, *S_T_* = 1.5279. (**f**) Latitude and longitude arraying form: 20°, 15°, *S_T_* = 2.6873. (**g**) Latitude and longitude arraying form: 20°, 30°, *S_T_* = 1.8247.

On the basis of the above numerical simulation results, the target surface could be divided into different regions, and the number of particle trajectories passing through each region could also be calculated. By comparing the distribution of the number of particle trajectories in each region, the uniformity of the distribution of particle trajectories on target surface could be explored accas outlined below. Firstly, all positions of the particle trajectories on the target surface were obtained on the basis of the numerical simulation results. Then, the target surface was divided into different regions, as shown in Figure 3a, and the number of particle trajectories passing over each region was counted, which could be used to calculate the ratio of the number of particle trajectories in each region to its area, i.e., *S_i_* (*I* = 1, 2, …, *N*). Finally, the standard deviation (*S_T_*) of all the values of *S_i_* could be obtained. Thus, *S_T_* was applied to quantitatively evaluate the uniformity of particle trajectories on the target surface, where a smaller value of *S_T_* indicates a better distribution of particle trajectories on the target surface [26].

Figure 3b–g show the distributions of *S_i_* in different regions and the associated *S_T_* with respect to different arraying forms of diamonds on the lapping plate considered in this study. For the fabrication of the surface microstructure on the bipolar plate, it is required that the morphology of the machined target surface should be regular and evenly distributed with the particle trajectories. It can be seen from Figure 3e that the fluctuation variation of *S_i_* from region 60 to 200 was stable within the range of 1 to 3, indicating that the particle trajectories in these regions were evenly distributed. By comparing with the other results shown in Figure 3, this fluctuation variation of *S_i_* was the smallest, and it is interesting to note that the value of *S_T_* was also the smallest. Therefore, it can be deduced that the arraying form of diamonds on the lapping plate shown in Figure 2d presented the best uniformity on the target surface in the fixed abrasive lapping of bipolar plate considered in this study.

## 5. Assessment of Fractal Characteristics in Particle Trajectories on Target Surface

In natural systems, e.g., the trunk, leaf venation, and blood circulation system of plants used to transport substances with the assistance of uniformly distributed microtubules, the flow field structures of these microtubules can be automatically adjusted according to the growth, divergence, or degradation of living conditions. These structures generally have fractal characteristics and can realize the intelligent and biological transmission of substances. Thus, it is necessary to assess the fractal characteristics of particle trajectories on the target surface with respect to the different arraying forms of diamonds on the lapping plate considered in this study, which can be used to optimize the design of a fixed abrasive lapping plate for the fabricating bipolar plate of PEMFCs.

Fractal theory is widely used in fractal image extraction, processing, and fractal dimension calculation. For the study of fractal characteristics, the fractal dimension is commonly used to measure the unevenness, complexity, or convolution degree of the application, in which the box-counting dimension has been extensively used in practice because of its clear concept, easy programming calculation, and good applicability [27,28]. Since the particle trajectories on the target surface can be directly and visually observed from Figure 2 and Figure 3, and these figures are indeed digital images possessing the characteristics of quantization and discretization, the box-counting dimension is a practical calculation method for the study of fractal characteristics of digital images, and the value of the box-counting dimension, *p*, can be taken from

(3) $$p=\underset{\delta \to 0^{+}}{\lim}\frac{\log N_{\delta }(F)}{-\log\delta }$$

**Figure 4:**
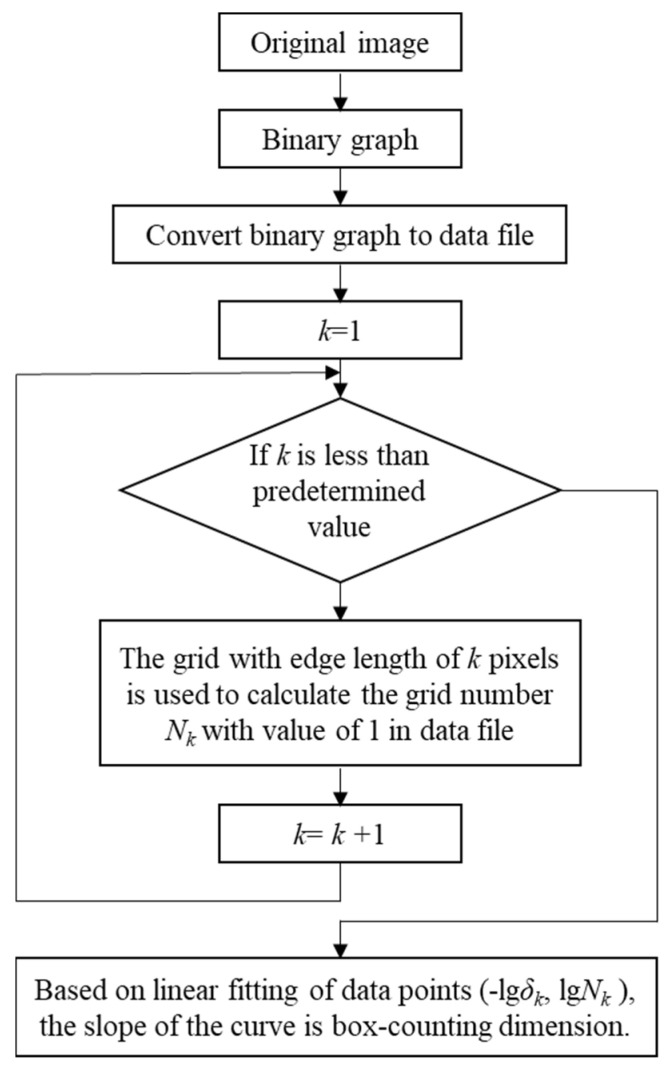
The calculation flow chart of the box-counting dimension.

Since the fractal characteristics are generally obtained from the digital images, during the calculation of the box-counting dimension, the image needs to be converted to a binary graph, where *δ* represents the size of the grid in the binary graph, and *N_δ_* (*F*) is the number of grids containing fractal feature pixels after meshing the binary graph according to the size of *δ*. The simulation results in Figure 2 were taken from the range of *x* [−90, 90] and *y* [–90, 90], such that when the box-counting dimension of the trajectory image was calculated, the size of the grid in the binary graph was less than or equal to 90, and the associated parametric programming calculation was carried out according to the flow chart as shown in Figure 4.

**Figure 5:**
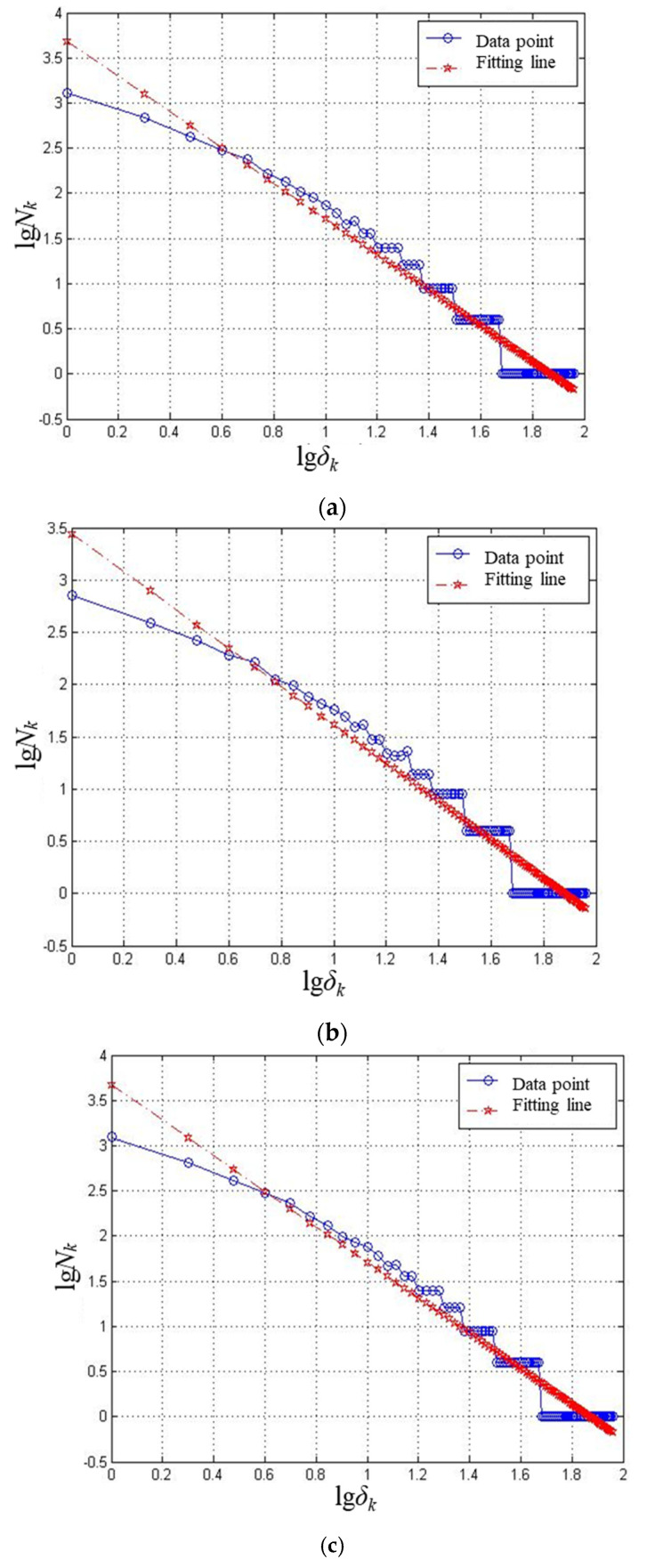

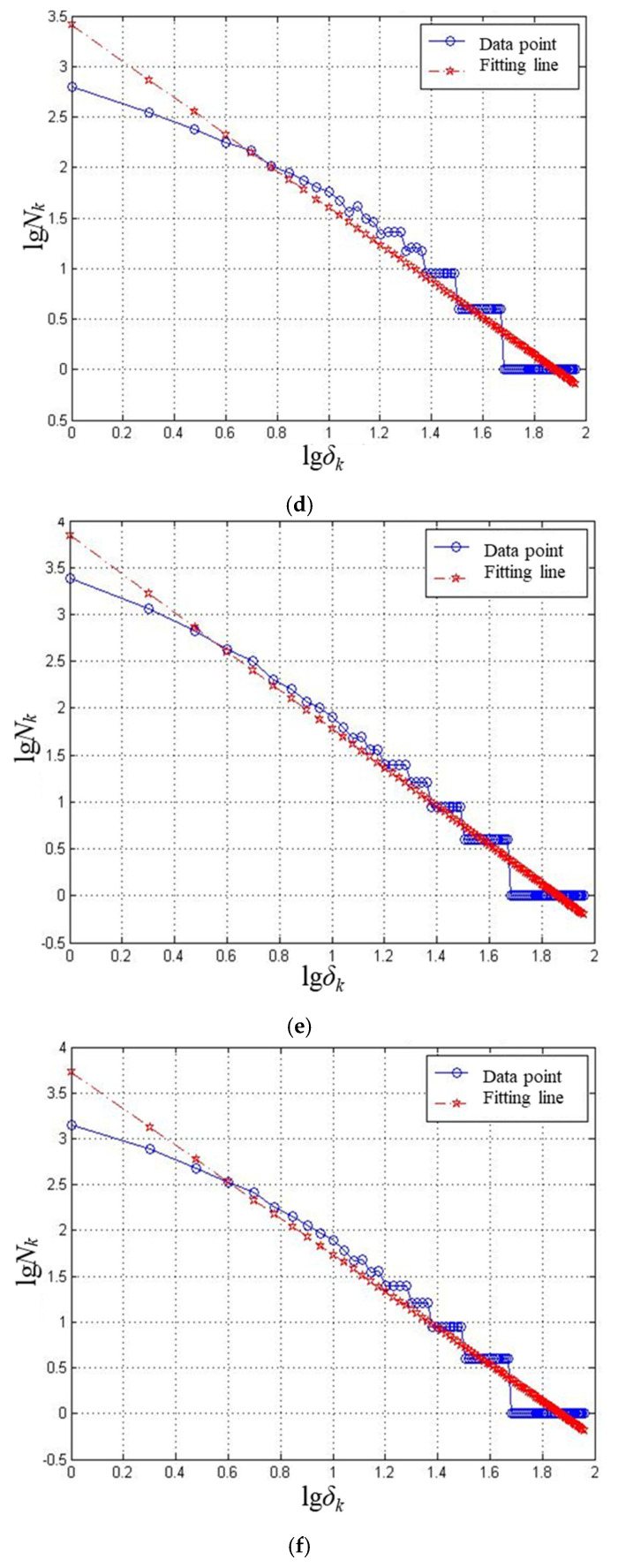
The linear fitting line of data points (−lg*δ_k_*, lg*N_k_*) from particle trajectories on the target surface for calculating the box-counting dimension, *p*, with respect to different arraying forms of diamonds on the lapping plate. (**a**) Circular arraying form: type-1, *p* = 1.9686. (**b**) Circular arraying form: type-2, *p* = 1.8303. (**c**) Latitude and longitude arraying form: 40°, 15°, *p* = 1.9611. (**d**) Latitude and longitude arraying form: 40°, 30°, *p* = 1.8121. (**e**) Latitude and longitude arraying form: 20°, 15°, *p* = 2.0639. (**f**) Latitude and longitude arraying form: 20°, 30°, *p* = 1.9930.

As shown in Figure 5, from the box-counting dimension fitting curve of the simulation trajectories, it can be seen that these trajectories all had fractal characteristics. The value of the box-counting dimension represents the density of trajectories, which means that a denser curve distribution indicates a higher value of the box-counting dimension. The surface microstructure of a bipolar plate not only requires uniform distribution of microchannels, but also requires a reasonable surface opening rate; hence, the fixed abrasive machining trajectories cannot be too dense, and the reasonable density should be controlled to ensure the opening rate. The results in Figure 5 show that the value of the box-counting dimension in Figure 5d was the lowest, which demonstrates that the arraying form of diamonds on the lapping plate shown in Figure 2d could obtain the lowest density in the fixed abrasive machining trajectories on target surface, which agrees with the results of the trajectory uniformity study. Therefore, through an analysis of the uniformity and fractal characteristics, it was found that diamonds arranged on the lapping plate, according to the distribution of the latitude and longitude with an angle of 30° and a gap of concentric circles of 40 mm with a minimum radius of 70 mm and a maximum radius of 190 mm, could be used to obtain the best uniformity and fractal characteristics in the fixed abrasive lapping of bipolar plates considered in this study.

In summary, the fixed abrasive lapping technology proposed in this paper was investigated and proven to be an effective and efficient way for fabricating surface microstructures on a bipolar plate with fractal characteristics, which can be employed as the flow field in a bipolar plate to realize intelligent energy trans-mission and obtain high-efficiency PEMFCs. Moreover, by properly designing the fixed abrasive lapping plate and using different types of abrasives, this technology can be used to fabricate complicated surface microstructures on solar cells for improving the photoelectric conversion efficiency. However, the limitations of this technology should be noted, such as how to quantitively control the dimensions of the microstructures and how to optimize the processing parameters by considering the proper genetic algorithm [29]; hence, future work should be conducted to address these issues.

## 6. Conclusions

The fixed abrasive lapping technology was found to be an avenue for the machining of surface microstructures on bipolar plates with fractal characteristics in this paper, and the main contributions of this paper can be summarized as follows:

- The kinematics of the fixed abrasive lapping process was first developed to explore the particle trajectories moving on the target surface by considering the different arraying forms of diamonds on the lapping plate, and then it was found from numerical simulations that the latitude and longitude distribution of diamonds on the lapping plate would result in a petal-shaped distribution of particle trajectories on the target surface with fractal characteristics, thus resulting in fixed abrasive lapping of bipolar plates with deterministic surface microstructures.
- A quantitative investigation was also carried out by analyzing the ratio of the number of particle trajectories in the divided region on the target surface to its whole area and the associated standard deviation, and it was found that the arraying form of diamonds on the lapping plate with latitude and longitude of 40°, 30° presented the best uniformity on the target surface in the fixed abrasive lapping of bipolar plates.
- The surface microstructure of a bipolar plate not only requires uniform distribution of microchannels, but also requires a reasonable surface opening rate; hence, the fixed abrasive machining trajectories cannot be too dense. Thus, the assessment of fractal characteristics in particle trajectories on the target surface was conducted using the box-counting dimension, and it was found that the arraying form of diamonds on the lapping plate with latitude and longitude of 40°, 30° showed the lowest density in the fixed abrasive machining trajectories on the target surface, which agreed with the results of the trajectory uniformity study.

Therefore, through an analysis of uniformity and fractal characteristics, it was found that diamonds arranged on the lapping plate, according to the distribution of latitude and longitude with an angle of 30° and a gap of the concentric circles of 40 mm with a minimum radius of 70 mm and a maximum radius of 190 mm, could be used to obtain the best uniformity and fractal characteristics in the fixed abrasive lapping of bipolar plates considered in this study.

## Data Availability

The data presented in this study are available on request from the corresponding author.
